# Exploring Impacts of Hyper-Branched Polyester Surface Modification of Graphene Oxide on the Mechanical Performances of Acrylonitrile-Butadiene-Styrene

**DOI:** 10.3390/polym13162614

**Published:** 2021-08-06

**Authors:** Xuebing Chen, Shulai Lu, Chunfu Sun, Zhenbiao Song, Jian Kang, Ya Cao

**Affiliations:** 1 State Key Laboratory of Polymer Materials Engineering, Polymer Research Institute of Sichuan University, Chengdu 610065, China; cxb451258932@gmail.com (X.C.); caoya@scu.edu.cn (Y.C.); 2 Synthetic Resin Factory of Jilin Petrochemical Company, PetroChina, Jilin 132021, China; jh_suncfu@petrochina.com.cn (C.S.); jh_szhenb@petrochina.com.cn (Z.S.); 3 PetroChina ABS Resin Technology Center, Jilin 132021, China

**Keywords:** graphene oxide, hyper-branched polyester, acrylonitrile-butadiene-styrene, mechanical performance

## Abstract

In this manuscript, the graphene oxide (GO) was modified by hyper-branched polyester (HBP). The effects of GO or modified GO (HBP-m-GO) on the mechanical performance and wearing properties were investigated. The results of X-ray photoelectron spectra (XPS), Fourier transform infrared spectroscopy (FT-IR), and transmission electron microscopy (TEM) revealed the successful grafting of HBP onto GO. The thermogravimetric analysis (TGA) indicated that the graft amount of HBP is calculated to be 9.6 wt%. The GO or HBP-m-GO was added into acrylonitrile-butadiene-styrene copolymer (ABS) to prepare the ABS/GO composites. The mechanical properties and wear performance of the composites were studied to comparatively study the impact of GO modification on the properties of the composites. The results revealed that the addition of GO has a significant effect on the mechanical properties of ABS, and when HBP-m-GO was added, the elastic modulus and tensile strength of ABS/HBP-m-GO increased evidently compared with ABS/GO. The tensile strength increased from 42.1 ± 0.6 MPa of pure ABS to 55.9 ± 0.9 MPa, up to 30%. Meanwhile, the elongation at break was significantly higher than ABS/GO to 20.1 ± 1.3%, slightly lower than that of pure ABS. For wear performance, the addition of raw GO decreased the friction coefficient, and when the HBP-m-GO was added, the friction coefficient of the ABS/HBP-m-GO dropped more evidently. Meanwhile, the weight loss during the wear test decreased evidently. The related mechanism was discussed.

## 1. Introduction

The acrylonitrile-butadiene-styrene (ABS) copolymer is a kind of plastic with excellent comprehensive properties [1,2]. The presence of high polar polyacrylonitrile component and rubber phase, as well as its unique multiphase composite structure, micro morphology and molecular structure endow it with good surface gloss, excellent mechanical properties, easy surface spraying, and electroplating characteristics [3]. Therefore, ABS resin is widely used in electronic and electrical appliances, automobile and other daily necessities, and industrial products manufacturing. However, the friction and wear resistance of ABS resin is limited and the friction coefficient is large [4]. On the other hand, it is urgent to have better mechanical properties [5]. Therefore, adding nano fillers into the ABS resin so as to further improve its mechanical and wear properties is considered to be an effective method, and also a research hotspot in related fields [6,7,8,9].

Graphene is a two-dimensional sheet material, with extremely thin thickness and high specific surface area [10,11,12], excellent electrical properties, thermal conductivity, and mechanical properties [13,14,15]. As a chemical precursor of graphene, graphene oxide (GO) can have some extent of attractive properties of graphene after chemical or thermal reductions [14,16,17]. GO also has a large number of reactive groups such as hydroxyl group, carboxyl group, and epoxy group distributed on its surface, which is easy for further organic modification, and is an ideal reinforcing material for ABS resins [18,19,20]. In order to improve the compatibility between GO and ABS resins and improve the dispersion uniformity of GO in ABS resins, it is a common method to use reactive groups on the GO surface to graft modification of macromolecules [21,22,23,24].

In recent years, the addition of derivatives of graphene into ABS have been extensively investigated. Wilkie C et al. [1] prepared composites by melt blending various graphites (virgin graphite, expandable graphites, and expanded graphite) with polystyrene (PS) and its copolymers (ABS and high-impact polystyrene (HIPS)), and comparatively studied the roles of graphites in enhancing the properties of the composites. Kharrat M et al. [2] systematically studied the impact of 0–7.5 wt% graphite particles in the mechanical and tribological properties in ABS composites, and observed a significant decrease of elastic modulus and failure strain, improvement of friction behavior and anti-wear abilities after the addition of graphite. They suggested that graphite strengthens the wear resistance of ABS composites and effectively reduces its adhesive and ploughing wear, and enhances the formation of a third body with better quality on the sliding stripe. Raza M et al. [8] prepared thermally reduced graphene oxide (TRGO)/ABS composites, and comparatively studied the effects of pristine graphite and TRGO on the mechanical and thermal properties of composites. They reported that the combined solution and melt mixing technique can significantly improve dispersion of TRGO in the ABS matrix. Kar K et al. [3] studied the effects of 0–40 vol% graphite flakes (GFs) in tensile, flexural, impact, hardness, and thermal conductivity performances of ABS/GFs composites, and reported that the GF-reinforced ABS composite with improved thermal conductivity, heat stability, viscoelastic behavior, and flexural modulus can be a promising as well as a suitable composite material for making various electronic and electrical accessories including bipolar plates for fuel cell applications. Hassan A et al. [5] prepared graphene nanoplates reinforced polycarbonate/ABS composites, and studied structural, morphological, mechanical, and thermal properties of the nanocomposites, claiming that the addition of GNP improved the flexural and tensile properties without the loss of extensibility and good thermal properties. Pal K et al. [7] enlightened the effect of three different derivatives of graphene on mechanical, thermal, and rheological properties of ABS composites prepared via the melt-mixing technique, i.e., GO, chemically reduced graphene oxide (rGO-C), and thermally reduced graphene oxide. They noticed that rGO-C acts as a better reinforcement in ABS than other derivatives. Khan N et al. [6] comparatively studied the reinforcement effects of two-dimensional few layer graphene (FLG) and one-dimensional multi-walled carbon nanotubes (MWCNT) in ABS using the tensile testing machine. To obtain the desired improvement of properties of the composites, one key factor is to improve the dispersion of the filler in the ABS matrix. However, current works mainly focus on the sample preparation method as well as the type and concentration of the filler. The impact of functional modification of the filler on the properties of the ABS composites is still urgent to be studied.

The hyper-branched polyester has a high degree of branching and a large number of hydroxyl terminated [25,26], low viscosity, high solubility, and high functionality, as well as has a very good compatibility with the ABS resin [27,28,29]. At the same time, it has a high reactivity with GO. It can be used as the surface graft of GO to further improve the compatibility between GO and ABS resins, which has not been reported before.

In this study, GO was modified by hyper-branched polyester (HBP) and then added into ABS resins to prepare the ABS/GO composites. The effects of hyper-branched polyester modification on the mechanical performance and wear properties of ABS/GO composites were studied.

## 2. Experimental Section

### 2.1. Materials and Sample Preparation

#### 2.1.1. Materials

ABS granules (tradename 0215H, MFI_2.16_ = 3.1 g/10 min) were kindly provided by Jilin Petrochemical Company, PetroChina Co. Ltd., Jilin, China. The hyper-branched polymer with the tradename of BOLTORN H202 (molecular weight = 600 g/mol, hydroxyl value = 550 mg KOH/g polymer) was purchased from Shanghai Bio Biotechnology Co., Ltd., Shanghai, China. *N*,*N*-dimethylformamide (DMF), analytical grade, were purchased from Sinopharm Chemical Reagent Beijing Co., Ltd., Beijing, China. The graphene oxide (GO), tradename N002-PDE, was purchased from Guangzhou Angstron graphene Technology Co. Ltd., Guangzhou, China. The average thickness of GO is lower than 1 nm, the lateral size is 0.5 and 1.5 micrometers, and the content of O atom is higher than 20%. All of the materials were used as received.

#### 2.1.2. Preparation of Surface Modified GO

**Scheme 1:**
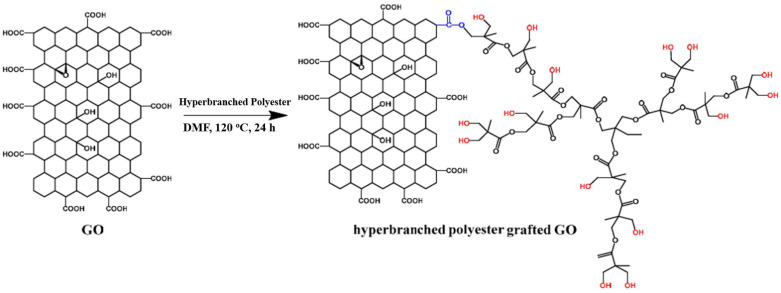
Illustration of the reaction between GO and hyper-branched polyester.

The illustration of the reaction between GO hand HBP is shown in Scheme 1. The hyper-branched polyester HBP and GO were dissolved in DMF at room temperature, and then irradiated using an ultrasound for 1 h. After that, they were heated up to 120 °C and reacted under magnetic stirring for 24 h. After that, they were cooled to room temperature slowly and filtered using the PVDF filtration membrane with the average pore diameter of 0.22 μm. The product was firstly washed by acetone and filtered several times to eliminate the unreacted HBP and extra solvent. Each time, the washed acetone was taken for the FT-IR analysis, and the washing is finished until the washed acetone has the same FT-IR spectra compared with pure acetone. The collected material was then heated under a vacuum drier at 80 °C for 24 h to obtain the final product, which was denoted as HBP-m-GO.

#### 2.1.3. Preparation of ABS/GO Composites

The composites were prepared by melt blending ABS with GO or HBP-m-GO at 200 °C for 10 min using a Brabender Plasticorder, Brabender Technologie, GMBH & CO. KG, Duisburg, Germany. The weight percentage of GO or HBP-m-GO was selected as 1.5% of the composites.

### 2.2. Characterization

#### 2.2.1. Fourier Transform Infrared Spectroscopy (FT-IR)

The sample was scanned by Nicolet IS50 FT-IR (Thermo Fisher Scientific Corp., Waltham, MA, USA) with a spectral resolution of 4 cm^−1^ and test range of 400–4000 cm^−1^. The sample was prepared by the KBr tablet pressing method [30,31].

#### 2.2.2. Transmission Electron Microscopy (TEM)

The transmission electron microscopy (TEM, Tecnai G2 F20, FEI Corp., Hillsboro, OR, USA) was used.

#### 2.2.3. Thermogravimetric Analysis (TGA)

The samples were heated to 800 °C at 20 °C/min under the protection of N_2_ and after equilibrium at 30 °C. The values were observed by the TG209F1 thermal analyzer (Netzsch Corp., Selb, Germany).

#### 2.2.4. X-ray Photoelectron Spectra (XPS)

The X-ray photoelectron spectra was carried out by the ESCALAB 250 photoelectron spectrometer (Thermo Fisher Scientific Corp., Waltham, MA, USA) with Al Kα (1486.6 eV) as the X-ray source set at 150 W and a pass energy of 30 eV for high resolution scan [32,33,34].

#### 2.2.5. Mechanical Properties

Tensile tests were performed on an INSTRON universal tensile machine equipped with 5 KN load cell and 25 mm displacement extensometer (Instron Corporation, Norwood, MA, USA) [35,36,37]. The crosshead speed for the tensile test was set at 50 mm/min. All of the tests were carried out at room temperature (25 ± 2 °C). Five to eight samples from each sample were tested and the averaged values were obtained [38,39,40]. The geometry of the samples was according to an international tensile testing norm AFNOR NF T 51-034.

#### 2.2.6. Wear Properties

Friction and wear tests were carried out using a reciprocating sliding tribometer connected to a computer monitoring the friction coefficient [41,42]. Tests were performed using a rectangular 30 × 10 × 20 mm^3^ composite sample sliding against a 35 mm diameter high chromium steel ball under a constant normal load *F_n_* = 17.16 N. The steel ball was kept in stationary and the tangential cyclic motion was applied to the composite specimen using the crank system driven by an electric motor with an electronic speed regulator [9,43,44,45]. The ball-on-plate machine was set to run with a tangential motion amplitude of 5 mm at 1 Hz. The tests were performed at a temperature of around 25 °C and a relative humidity (RH) between 50–60%. Durations of sliding tests were fixed to 1000, 2500, 4000, 5500, 7000, 8500, and 10,000 cycles. At least four tests were performed for each set of conditions.

## 3. Results and Discussion

### 3.1. Characterization of the Modified GO

The microstructures of GO, modified GO, and HBP were investigated by XPS, FT-IR, TGA, and TEM.

#### 3.1.1. XPS

**Figure 1:**
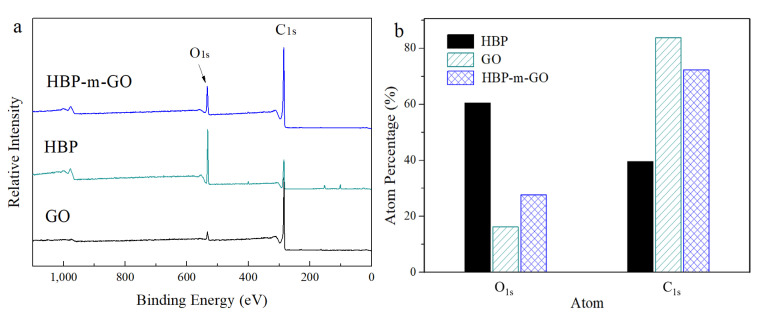
(**a**) XPS profiles of HBP, GO, and HBP-m-GO and (**b**) atomic percentages of O_1s_ and C_1s_.

**Figure 2:**
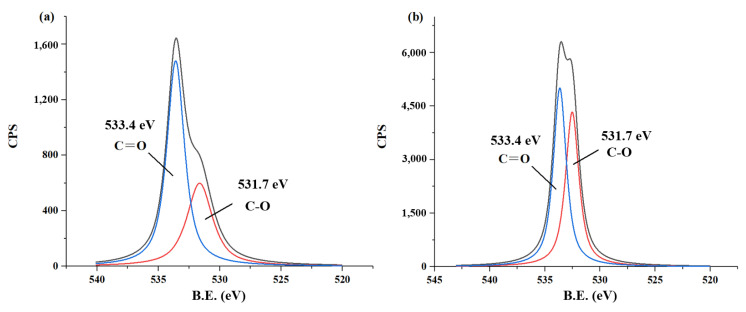
Detailed analysis of the O_1s_ profile of XPS results of (**a**) GO and (**b**) HBP-m-GO.

The XPS spectrum of neat GO, HBP-m-GO, and HBP are shown in Figure 1. From Figure 1, the detailed analysis on the C_1s_ and O_1s_ were carried out, the peak fitting results have been shown in Figure 1b and Figure 2 and Table 1.

As can be seen from the XPS results, the O and C element contents of HBP, GO, and HBP-m-GO are greatly different. The C atom content of GO was 83.7%, which was higher than that of HBP (39.5%) and HBP-m-GO (72.3%). On the other hand, the content of O element in GO is 16.7%, which is lower than that in HBP-m-GO (27.7%) and HBP (60.5%). The ratio *n*(O)/*n*(C) of O element and C element was calculated, and it can be found that the *n*(O)/*n*(C) of GO is 0.19, and the *n*(O)/*n*(C) of HBP is 1.53. After the grafting reaction, the *n*(O)/*n*(C) of HBP-m-GO increased from 0.19 to 0.38, indicating that the content of oxygen element was significantly increased. As can be seen from the detailed analysis in Figure 2 and Table 1, after grafting HBP, the percentage of C=O of HBP-m-GO decreases compared with neat GO, while the percentage of C–O of HBP-m-GO increases. In summary, all of the XPS results above confirmed the success of the grafting reaction.

#### 3.1.2. FT-IR

**Figure 3:**
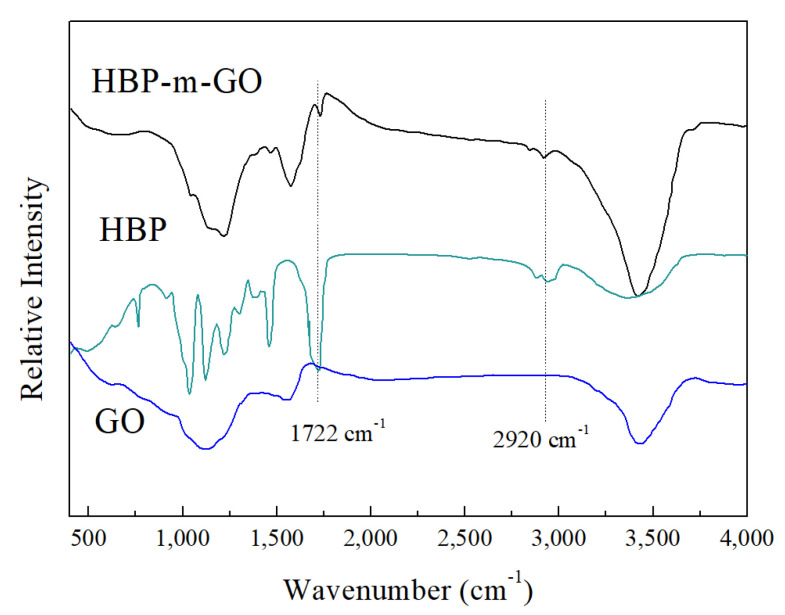
FT-IR spectrum of HBP, GO, and HBP-m-GO.

Figure 3 is the infrared spectrum of the sample. For HBP, the peak at 3420 cm^−1^ corresponds to the vibration of –OH group, while signals at the wavenumbers of 2920 and 2888 cm^−1^ represent the asymmetric and symmetric vibrations of –CH_3_ and –CH_2_, respectively [46,47,48]. The peaks at the wavenumber of 1722 cm^−1^ are the stretching vibration peak of carbonyl [49,50]. 

On the other hand, by comparing the infrared spectra of GO and HBP-m-GO, it can be found that, compared with pure GO, new characteristic peaks appear on the infrared spectra of HBP-m-GO, which are located at 2921, 2888, and 1734 cm^−1^ [51,52,53]. These three absorption peaks correspond to the asymmetric and symmetric vibrations of –CH_3_ and –CH_2_, and stretching vibration peak of carbonyl, respectively, suggesting that the HBP is successfully grafted onto GO. Obviously, these three peaks all come from HBP, indicating that HBP was successfully grafted onto GO.

#### 3.1.3. TGA

**Figure 4:**
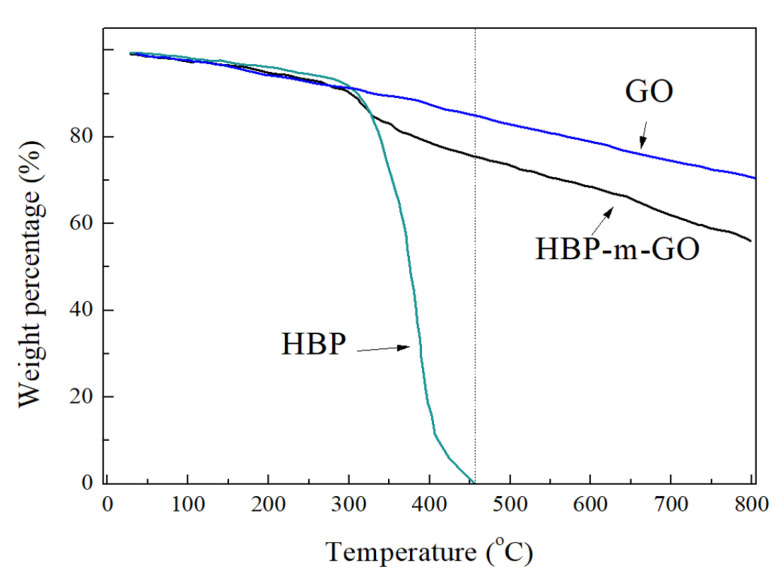
TGA results of HBP, GO, and HBP-m-GO.

Figure 4 is the TGA curve of the sample. It can be seen that the heat resistance of HBP is poor, and its weight loss takes place in the range of about 300–450 °C, and almost completely decomposes when the temperature is higher than 450 °C. GO has the least thermal weight loss and the best thermal stability. However, HBP-m-GO exhibits a certain degree of weight loss in the range of 300–457 °C, which is caused by the grafting of HBP onto it. It can be seen from Figure 4 that, at the temperature of 457 °C (indicated by a short dash line in the figure), the residue weights of GO and HBP-m-GO are 85.1 and 75.5 wt% respectively, and the graft amount of HBP is calculated to be about 9.6 wt%.

#### 3.1.4. TEM

**Figure 5:**
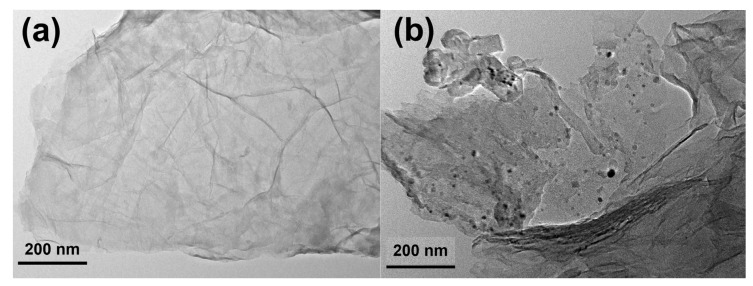
TEM results of (**a**) neat GO and (**b**) HBP-m-GO.

To directly observe the morphology of the fillers, TEM was performed as shown in Figure 5. Clearly, compared with neat GO, many dark dots can be observed from the TEM image of HBP-m-GO, which might be the evidence for the successful grating of HBP onto GO.

### 3.2. Mechanical Performance

**Figure 6:**
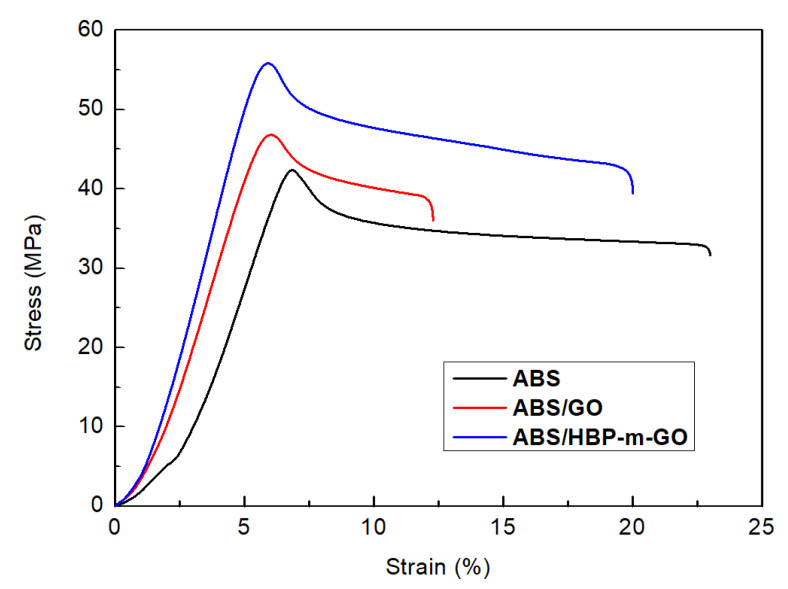
Stress-strain curves of ABS, ABS/GO, and ABS/HBP-m-GO.

Figure 6 shows the typical tensile stress-strain curves for the neat ABS and its composites. It is shown that the typical yield behavior occurs in stress strain curves for the composites with GO or HBP-m-GO. The addition of GO or HBP-m-GO reduces the tensile tendency of the material, meaning that the greater tensile stress is needed to stretch and yield. It is clear from the results in Figure 6 and Table 2 that when the unmodified GO is added, the tensile strength of ABS/GO increased from 42.1 ± 0.6 MPa of pure ABS to 42.8 ± 1.3 MPa, while the elastic modulus increased from 1.15 ± 0.03 GPa to 1.21 ± 0.05 GPa. Meanwhile, the elongation at break decreased drastically from 23.2 ± 1.2% of pure ABS to 11.3 ± 2.0%. These trends indicate that the addition of GO has a significant effect on the mechanical properties of ABS, which is mainly attributed to the weak interface interaction between ABS and GO and the lack of chemical bonds.

Interestingly, when the modified GO was added, the elastic modulus and tensile strength of ABS/HBP-m-GO increased evidently. The tensile strength increased from 42.1 ± 0.6 MPa of pure ABS to 55.9 ± 0.9 MPa, up to 30%. Meanwhile, the elongation at break of the sample was significantly higher than ABS/GO, to 20.1 ± 1.3%, slightly lower than that of pure ABS resin. This might be attributed to the significantly enhanced interaction between the ABS matrix with GO after surface grafting modification. During stretching, the area of weak interfaces between ABS and HBP-m-GO significantly decreased, resulting in the maintaining of elongation at break at a higher level. Meanwhile, the elastic modulus and tensile strength are significantly enhanced.

### 3.3. Wear Performance

**Figure 7:**
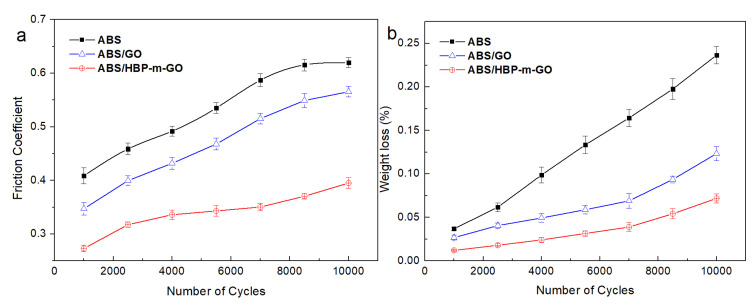
(**a**) Friction coefficient and (**b**) weight losses (%) versus number of sliding cycles for ABS and ABS/GO composites.

Figure 7a shows the relationship between the coefficient of friction and the sliding cycles of pure ABS and ABS/GO composites. It is shown that for all the samples, the coefficient of friction increases monotonically with the number of sliding times. Pure ABS exhibits a high coefficient of friction (0.41) after 1000 sliding cycles. As the number of cycles increases, the friction coefficient gradually increases, after 10,000 cycles, the pure ABS specimen wears out severely and the friction coefficient increases significantly to 0.62. Compared with pure ABS, the addition of raw GO decreases the friction coefficient compared to pure ABS, a rule consistent in 10,000 cyclic tests. On the other hand, when the modified GO is added, the friction coefficient of the ABS/HBP-m-GO drops further, at 0.27 after 1000 sliding cycles, and after 10,000 cycles, at 0.395. This shows that the addition of the modified GO significantly reduces the coefficient of friction of the ABS. 

On the other hand, it is well known that the worse the wear resistance, the greater the wear losses. From the curve of the sample mass loss-cyclic friction number in Figure 7b, the pure ABS resin has the lowest wear resistance and the largest mass loss in the friction cycle test, and after 10,000 cyclic frictions, it reaches 0.24%. After adding the unmodified GO, the wear amount is greatly reduced, indicating that the GO added improves the wear resistance of the ABS resin. When the modified GO is added, the wear amount of the composite decreases further. After 10,000 cyclic frictions, the wear only reaches 0.07%, which is 29% of the same condition as pure ABS. The above results show that the surface modification of the GO can significantly improve the friction resistance of the ABS resin, possibly since the surface modification of the GO makes it a more uniform dispersion in the ABS resin and has a stronger interface binding force with the ABS matrix.

**Figure 8:**
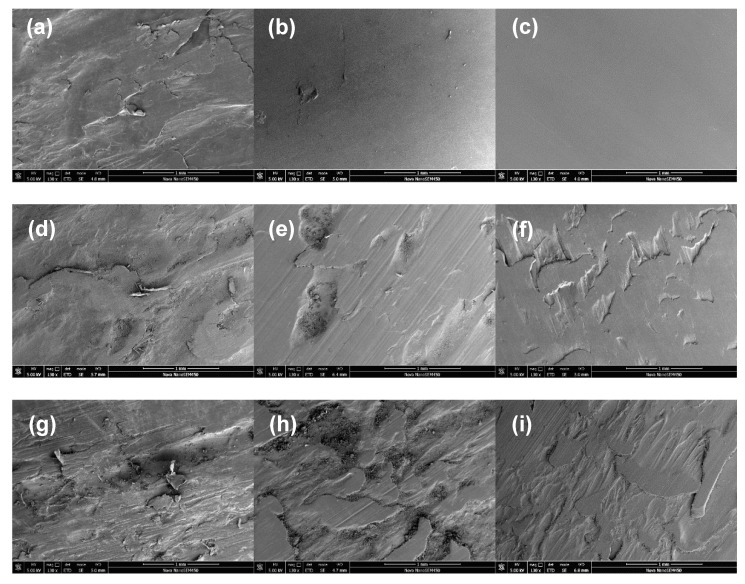
SEM images of the worn surface morphological features of pure ABS, ABS/GO and ABS/HBP-m-GO after (**a**–**c**) 1000, (**d**–**f**) 4000 and (**g**–**i**) 10,000 sliding cycles, respectively.

The SEM observation was used to investigate the effect of the fillers on the worn surface morphological features of ABS after 1000, 4000, and 10,000 sliding cycles (Figure 8).

Morphologies of the worn surfaces formed after 1000 sliding cycles are shown in Figure 8a–c. For the neat ABS (Figure 8a), a large area of surface deformation can be seen, characterized by plastic deformation and plough constitute predominant surface modification, indicating that the adhesive wear mechanism is activated. Meanwhile, the deformed area of ABS/GO is much smaller than the neat ABS, and almost no deformation can be seen from ABS/HBP-m-GO, which is in accord with the result in Figure 7.

At 4000 sliding cycles (Figure 8d–f), a similar trend can be observed. The surface of neat ABS is seriously worn, while the severity of the wear of ABS/HBP-m-GO is lowest. 

At 10,000 sliding cycles (Figure 8h–j), the seriously worn surface of neat ABS can be seen, characterized by severe peeling off and plastic deformation (Figure 8h), corresponding to its worse tribological properties. When raw GO was added, it can be seen that the worn surface is relatively smooth compared with the neat ABS, reflecting that the addition of GO benefits for wear reduction. However, it should be emphasized that some debris can be noticed for ABS/GO, which might be attributed to the limited compatibility between raw GO and ABS. For ABS/HBP-m-GO, its worn surface is obviously smoother compared with the neat ABS and ABS/GO and almost no debris can be seen, indicating the evidently enhancement of tribological properties, which might be attributed to the surface modification of GO and the strengthened compatibility between HBP-m-GO and the polymer matrix.

## 4. Conclusions

In this manuscript, the GO surface was modified by hyper-branched polyester and the ABS/GO and ABS/HBP-m-GO composites were prepared. The effects of GO or modified GO on the mechanical performance and wearing properties were investigated, as follows:

(1) The results of XPS, FT-IR, and TEM revealed the successful grafting of HBP onto GO. In addition, TGA indicated that the graft amount of HBP is calculated to be about 9.6 wt%.
(2) ABS/GO composites have been prepared, and the mechanical properties and wear performance were studied in detail. The results herein reviewed that the addition of GO has a significant effect on the mechanical properties of ABS, and when the modified GO was added, the elastic modulus and tensile strength of ABS/HBP-m-GO increased evidently. The tensile strength increased from 42.1 ± 0.6 MPa of pure ABS to 55.9 ± 0.9 MPa, up to 30%. Meanwhile, the elongation at break of the sample was significantly higher than ABS/GO, to 20.1 ± 1.3%, slightly lower than that of pure ABS resin. For wear performance, the addition of raw GO decreases the friction coefficient compared with pure ABS, and when the modified GO is added, the friction coefficient of the ABS/HBP-m-GO drops further, at 0.27 after 1000 sliding cycles, and after 10,000 cycles, at 0.395, revealing that the addition of the modified GO significantly reduces the coefficient of friction of the ABS. Meanwhile, the weight loss during the wear test decreases evidently. Finally, the morphological analysis was conducted and the related mechanism was analyzed.

## Figures and Tables

**Table 1:** Detailed analysis of the O_1s_ profile of XPS results of GO, HBP-m-GO, and HBP.

Type	Position (eV)	GO (%)	HBP-m-GO (%)	HBP (%)
C−O	531.7	37.0	47.3	63.1
C=O	533.4	64.0	52.7	36.9

**Table 2:** Mechanical parameters of ABS, ABS/GO, and ABS/HBP-m-GO.

Samples	Composition (wt%)			Elastic Modulus (GPa)	Tensile Strength (MPa)	Elongation at Break (%)
	ABS	GO	HBP-m-GO			
ABS	100	-	-	1.15 ± 0.03	42.1 ± 0.6	23.0 ± 1.2
ABS/GO	98.5	1.5	-	1.21 ± 0.05	46.8 ± 1.3	11.3 ± 2.0
ABS/HBP-m-GO	98.5	-	1.5	1.43 ± 0.04	55.9 ± 0.9	20.1 ± 1.3

## Data Availability

The data presented in this study are available on request from the corresponding author.
